# Uric Acid Levels in Overweight and Obese Children, and Their Correlation With Metabolic Risk Factors

**DOI:** 10.7759/cureus.70160

**Published:** 2024-09-25

**Authors:** Mohammed A AlAteeq, Abdallh Almaneea, Eyad K Althaqeb, Meshal F Aljarallah, Abdulazeez E Alsaleh, Malek A Alrasheed

**Affiliations:** 1 Family Medicine Department, Ministry of National Guard - Health Affairs, Riyadh, SAU; 2 Family Medicine, King Abdullah International Medical Research Center, Riyadh, SAU; 3 Family Medicine, King Saud Bin Abdulaziz University for Health Sciences, Riyadh, SAU; 4 Anesthesia, King Faisal Specialist Hospital and Research Centre, Riyadh, SAU; 5 Family Medicine and Primary Care, King Abdulaziz Medical City Riyadh, Riyadh, SAU; 6 Collage of Medicine, King Saud Bin Abdulaziz University for Health Sciences, Riyadh, SAU; 7 Primary Care, King Faisal Specialist Hospital and Research Centre, Riyadh, SAU

**Keywords:** hyperglycemia, hyperlipidemia, hyperuricemia, metabolic disease, pediatrics

## Abstract

Background: Obesity is an ongoing medical condition that continues to rise on a global scale. Numerous metabolic disorders, such as type 2 diabetes mellitus, hyperlipidemia, and hyperuricemia, are closely associated with obesity. This particular study aims to investigate the occurrence of hyperuricemia and its association with other metabolic factors among children and adolescents aged 6 to 14 years who are overweight or obese residing in Riyadh, Saudi Arabia.

Methods: In this research, a cross-sectional, descriptive, and analytical study was carried out on 339 children and adolescents. These participants were selected randomly from a list of patients who had sought medical care for overweight and obesity at the pediatric endocrinology, general pediatrics, and family medicine clinics in King Abdulaziz Medical City (KAMC), located in Riyadh, Saudi Arabia, in the period from January 2020 to January 2022. To gather the necessary data, the electronic medical records of the participating individuals were carefully reviewed, encompassing various relevant variables, including demographic characteristics, anthropometric measurements, serum uric acid levels, lipid profiles, and fasting blood sugar.

Results: Out of the total 339 participants, 48 (14.2%) were identified as overweight, while 291 (85.8%) were classified as obese. The study revealed that the overall prevalence of hyperuricemia among the participants was 54%. There was an increased risk of hyperuricemia associated with male gender and elevated levels of LDL and TG.

Conclusion: The significant prevalence of hyperuricemia among children and adolescents who are overweight or obese is evident. In order to improve control and management of this condition, it is crucial to prioritize the promotion of a healthy lifestyle among these individuals.

## Introduction

Obesity is a long-term medical issue marked by excessive fat accumulation, closely linked to various metabolic disorders. The World Health Organization (WHO) defines overweight and obesity in children aged 5 to 19 years as a BMI-for-age exceeding 1 standard deviation above the WHO Growth Reference median for overweight and more than 2 standard deviations above the median for obesity [1].

The global prevalence of obesity is increasing, impacting both adults and children. In 2019, the World Obesity Federation projected that by 2025, around 206 million children and adolescents aged 5 to 19 would be affected by obesity. Additionally, they estimated that this figure would grow to 254 million by 2030. Among the 42 countries expected to have over 1 million children with obesity by 2030, China was ranked highest, followed by India, the USA, Indonesia, and Brazil. Notably, only seven of these top 42 nations were classified as high-income countries [2].

Obesity and overweight rates among Saudi children have been increasing over the past decade. The most recent data published in 2019 indicated prevalence rates of 13.4% for overweight and 18.2% for obesity, based on a sample of 7,931 subjects aged 6 to 16 years [3].

Obesity is acknowledged as a significant risk factor for cardiovascular diseases, type 2 diabetes mellitus, metabolic syndrome, and related conditions. As the rates of juvenile obesity continue to escalate, the incidence of metabolic syndrome, also referred to as "syndrome X," increases correspondingly [3,4]. Numerous studies have indicated a connection between elevated serum uric acid (UA) levels and the onset of metabolic syndrome, along with a heightened risk of cardiovascular and renal diseases [5-7].

UA levels in children typically fluctuate based on age and sex, with a rise during puberty, reaching adult levels between ages 15 and 17 years [8].

Numerous international studies have shown a relationship between childhood obesity and elevated uric acid levels. One study involving 764 obese children in Brazil identified a significant linear trend of increasing uric acid levels alongside other cardiovascular risk factors, correlating with overweight and obese groups [9]. Another study conducted in Thailand, which included 689 secondary school students of both sexes, found a similar association [10].

In a Danish study involving 171 children aged 4 to 18 years with obesity, UA was positively linked to changes in BMI. UA levels declined in the 65 children who lost weight during the study, while they rose in the 23 children who gained weight throughout the trial. [11]. Similar findings have been reported as well in Spain [12], Taiwan [13], Turkey [14], and Romania [15]. Regionally, in Egypt, a study showed a significant correlation of UA with components of metabolic syndrome [16].

To our knowledge, there are no local studies examining hyperuricemia in children with obesity or overweight. The aim of this study is to assess the prevalence of hyperuricemia among overweight and obese children and adolescents in Riyadh, Saudi Arabia.

The aim of this particular study is to investigate the occurrence of hyperuricemia, and its association with other metabolic factors among children and adolescents aged 6 to 14 years with overweight or obesity residing in Riyadh, Saudi Arabia.

## Materials and methods

This study focused on a cross-sectional, descriptive, and analytical investigation involving children and adolescents aged 6 to 14 years who were diagnosed with overweight or obesity. The participants were selected randomly from patients who visited pediatric endocrinology clinics, general pediatrics clinics, and three family medicine clinics (Health Care Specialty Center (HCSC), King Abdulaziz City Housing (Iskan Yarmouk), and National Guard Comprehensive Specialized Clinic (NGCSC)) at King Abdulaziz Medical City (KAMC) in Riyadh, Saudi Arabia, between January 2020 and January 2022. Children with secondary obesity, hypertension, chronic liver and kidney diseases, malignancies, and those taking medications that could affect uric acid (UA) metabolism were excluded from the study.

The estimated sample size for this research was based on a reported 20% prevalence of hyperuricemia in obese children [17]. Utilizing the Raosoft Sample Size calculator with a 95% confidence level and a 5% margin of error, the calculated sample size was 246 participants. However, we included a total of 339 patients in our study. After receiving approval from the Institutional Review Board (IRB) at King Abdulla International Medical Research Center, we gathered data related to the study variables by reviewing the participants' electronic medical records. The study variables included demographic details (age and gender), anthropometric measurements (weight, height, and body mass index [BMI]), along with the most recent laboratory results for UA.

The primary outcome variable assessed in this study was the UA level. Abnormal UA levels were classified by age group, following the research conducted by W.D. Wilcox. For children aged 6 to 10 years (regardless of gender), UA levels exceeding 5.1 mg/dL (>303 mmol/L) were deemed abnormal. In the 11- to 14-year age group, abnormal UA levels were defined as above 5.5 mg/dL (>327 mmol/L) in males and above 5.4 mg/dL (>321 mmol/L) in females. Atherosclerotic cardiovascular risk factors included in this study were glucose and lipid profiles. Total cholesterol levels were considered abnormal for values exceeding 5.2 mmol/L, LDL for levels above 3.4 mmol/L, HDL for levels below 1 mmol/L, TG for levels above 1.1 mmol/L for ages 7-9 years, and 1.5 mmol/L for ages 10-14 years, and fasting blood sugar for levels exceeding 5.6 mmol/L.

Statistical analysis plan

Data was entered into an Excel sheet, cleaned, and imported into R software (version 4.2.2). Data normality was assessed using histograms and the Shapiro test. Descriptive statistics were used to summarize the data, with continuous variables presented as median and interquartile range, while categorical variables were presented as numbers and percentages. Pearson's Chi-squared, Fisher's exact, and Wilcoxon rank sum tests were used to evaluate hyperuricemia determinants. Multiple logistic regression was employed to determine the association between hyperuricemia and the demographics and atherosclerotic cardiovascular risk factors.

The study was approved by the Institutional Review Board (IRB) at King Abdullah International Medical Research Center (KAIMRC) under approval number IRB/1121/22 on June 20, 2022.

## Results

The study comprised 339 participants, with most aged 11-14 years (88%), and 51% were female. A majority of participants did not have a chronic disease (97%), and 98% of them are not on medication currently. The median weight was 77 kg, and the median height was 156 cm.

Regarding weight status, most participants were classified as obese (59%), followed by severely obese (27%), and overweight (13.8%) (Table 1).

**Table 1:** Demographic characteristics of the study participants 1n (%); Median (IQR: interquartile range)

Characteristic	N = 339^1^
Age	
11-14 years	299 (88%)
7-10 years	40 (12%)
Gender	
Female	174 (51%)
Male	165 (49%)
Chronic disease	
No	329 (97%)
Yes	9 (2.7%)
Unknown	1
Current medication	
No	332 (98%)
Yes	7 (2.1%)
Weight (Kg)	77 (66, 89)
Height (cm)	156 (151, 163)
Weight status	
Overweight	48 (13.8%)
Obese	200 (59%)
Severely obese	91 (27%)

**Figure 1:**
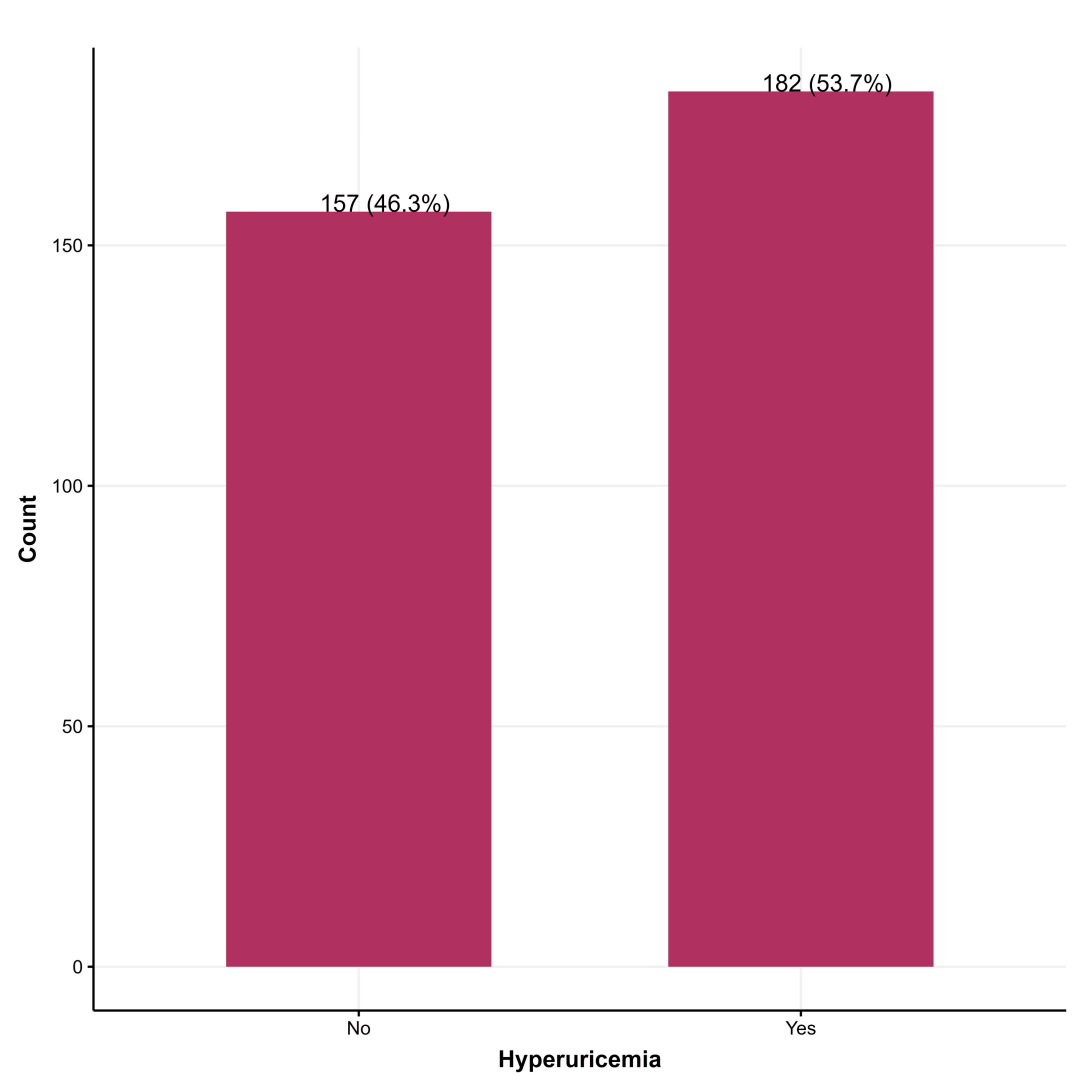
Serum uric acid levels among study participants

In this study, around 54% of participants had hyperuricemia (Figure 1).

The median total cholesterol (TC), low-density lipoprotein (LDL), high-density lipoprotein (HDL), triglycerides (TG), and fasting blood sugar (FBS) were 4.10 mmol/L, 2.81 mmol/L, 1.13 mmol/L, 0.92 mmol/L, and 5.00 mmol/L, respectively. 

Among participants, 8.6% had TC levels above the 95th percentile, 19% had LDL above it, and 12% had TG above it. About 26% of participants fell below the 10th percentile of HDL, while 74% were above it. Approximately 91% of participants had normal glycemic levels. The median serum uric acid level was 5.55 mg/dL (Table 2).

**Table 2:** Uric acid, lipid, and glucose profiles of the study participants (N = 339) IQR: Interquartile range, TC: Total cholesterol, LDL: Low density lipoprotein, HDL: High density lipoprotein, TG: Triglycerides, FBS: Fasting blood sugar

Characteristic	Median (IQR)
Serum uric acid (mg/dL)	5.55 (4.67, 6.63)
TC (mmol/L)	4.10 (3.70, 4.60)
TC Percentile	
<95th	310 (91%)
>95th	29 (8.6%)
LDL (mmol/L)	2.81 (2.36, 3.28)
LD. Percentile	
<95th	273 (81%)
>95th	66 (19%)
HDL (mmol/L)	1.13 (0.99, 1.28)
HDL Percentile	
<10th	87 (26%)
>10th	252 (74%)
TG (mmol/L)	0.92 (0.73, 1.18)
TG Percentile	
<95th	297 (88%)
>95th	42 (12%)
FBS (mmol/L)	5.00 (4.70, 5.30)
Hyperglycemia	
No	307 (91%)
Yes	32 (9.4%)

Being male (p<0.001) and obese (p=0.043) were linked to hyperuricemia. Higher LDL (p=0.05) and higher TG (p=0.007) were associated with hyperuricemia. Age and chronic disease status had no significant association with hyperuricemia (Table 3).

**Table 3:** Determinants of hyperuricemia ^1^Pearson's chi-squared test; Fisher's exact test; Wilcoxon rank sum test ^2^Median (IQR) TC: Total cholesterol, TG: Triglyceride, HDL: High density lipoprotein, LDL: Low density lipoprotein, FBS: Fasting blood sugar

Characteristic		Hyperuricemia		p-value^1^
		No (n= 157)	Yes (n=182)	
Age	11-14 years	133 (85%)	166 (91%)	0.065
	7-10 years	24 (15%)	16 (8.8%)	
Gender	Female	101 (64%)	73 (40%)	<0.001
	Male	56 (36%)	109 (60%)	
Chronic disease	Yes	4 (2.6%)	5 (2.7%)	>0.9
	Unknown	1	0	
Weight Status	Overweight	18 (11.4%)	30 (16.4%)	0.043
	Obesity	105 (66.8%)	95 (52%)	
	Severe obesity	34 (21.6%)	57 (31%)	
TC (mmol/L)^2^		4.08 (3.70, 4.56)	4.19 (3.70, 4.70)	0.4
TC Percentile	<95th	145 (92%)	165 (91%)	0.6
	>95th	12 (7.6%)	17 (9.3%)	
LDL (mmol/L)^2^		2.70 (2.32, 3.13)	2.89 (2.36, 3.32)	0.050
LDL Percentile	<95th	131 (83%)	142 (78%)	0.2
	>95th	26 (17%)	40 (22%)	
HDL (mmol/L)^2^		1.18 (1.01, 1.29)	1.09 (0.98, 1.26)	0.069
HDL Percentile	<10th	35 (22%)	52 (29%)	0.2
	>10th	122 (78%)	130 (71%)	
TG (mmol/L)^2^		0.86 (0.68, 1.12)	0.98 (0.79, 1.27)	0.007
TG Percentile	<95th	140 (89%)	157 (86%)	0.4
	>95th	17 (11%)	25 (14%)	
FBS (mmol/L)^2^		5.00 (4.70, 5.30)	5.00 (4.70, 5.40)	0.5

However, for gender, being male showed a stronger association compared to females in both univariate (odds ratio [OR]: 2.97, 95% confidence interval [CI]: 1.90-4.67) and multivariate (OR: 3.48, 95% CI: 2.16-5.67) analyses. Increases in LDL (multivariate: OR: 4.74, 95% CI: 1.98-15.3) and TG (multivariate: OR: 2.35, 95% CI: 1.16-5.03) were associated with a higher risk of hyperuricemia.

In contrast, increasing TC levels showed a lower risk of hyperuricemia (OR: 0.20, 95% CI: 0.06-0.54). Conversely, HDL levels and FBS levels did not demonstrate significant associations.

**Figure 2:**
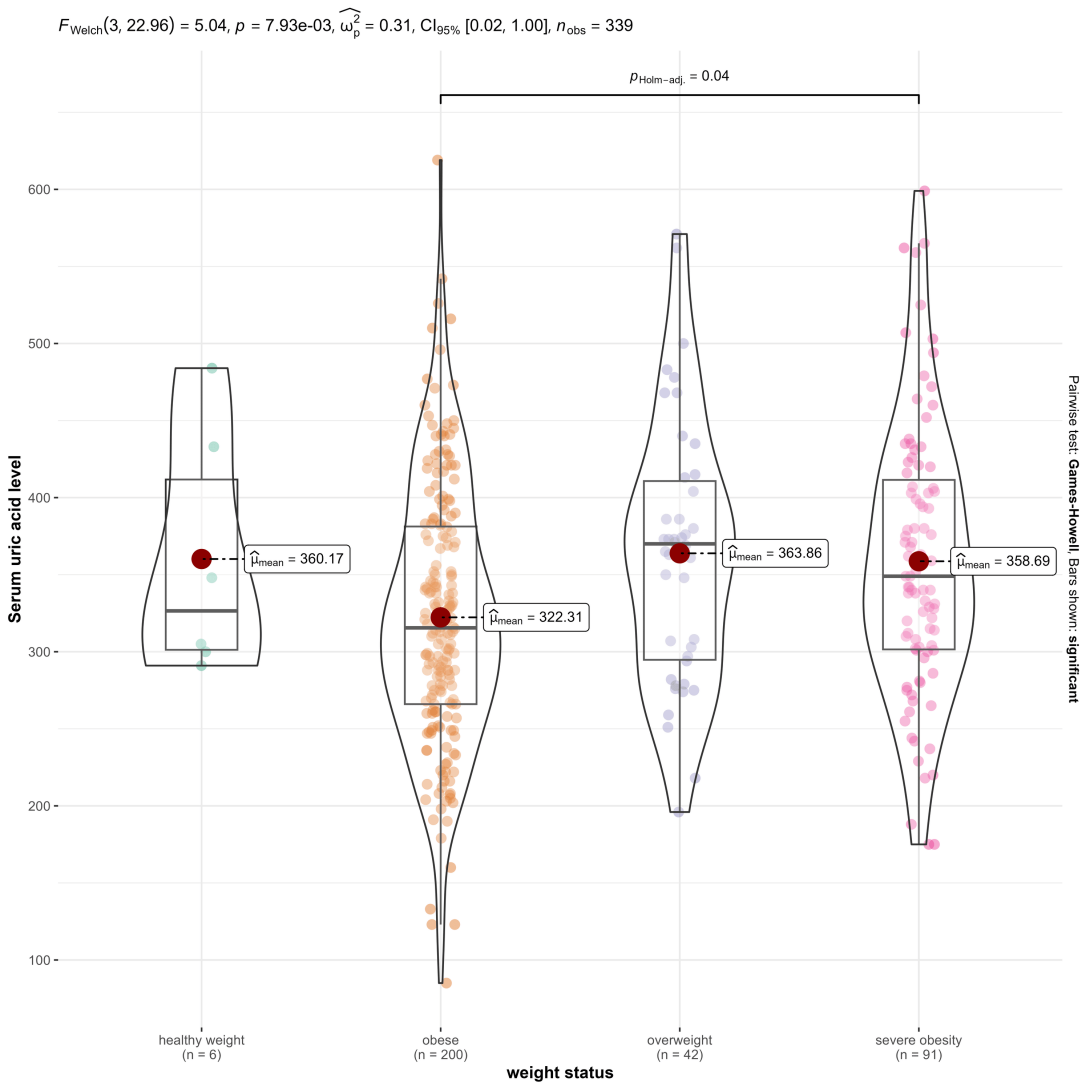
Association between BMI and mean serum uric acid (umol/L) CI: Confidence interval

The association of hyperuricemia with participant characteristics is shown in Table 4. That overall trend in Figure 2 suggests a higher uric acid level in heavier individuals. However, there is considerable overlap in the distributions of serum uric acid levels across the different weight status groups. This indicates that individual variability is significant, and not all individuals in a given weight category will have elevated uric acid levels. 

**Table 4:** Association of hyperuricemia with participants characteristic 1*p<0.05; **p<0.01; ***p<0.001 OR: Odds ratio, CI: Confidence interval, TC: Total cholesterol, TG: Triglyceride, HDL: High density lipoprotein, LDL: Low density lipoprotein, FBS: Fasting blood sugar

Characteristic	Univariate		Multivariate	
	OR	95% CI	OR	95% CI
Age				
11-14 years	—	—	—	—
7-10 years	0.53	0.27, 1.03	0.50	0.24, 1.02
Gender				
Female	—	—	—	—
Male	2.97***	1.90, 4.67	3.48***	2.16, 5.67
Chronic disease				
No	—	—	—	—
Yes	1.07	0.28, 4.39	2.93	0.57, 17.3
Weight Status				
Overweight	—	—		
Obese	0.50	0.25, 0.99		
Severely obese	0.93	0.43, 1.98		
TC (mmol/L)	1.11	0.83, 1.49	0.20**	0.06, 0.54
TC Percentile				
<95th	—	—		
>95th	1.24	0.58, 2.75		
LDL (mmol/L)	1.34	1.00, 1.80	4.74**	1.98, 15.3
LDL Percentile				
<95th	—	—		
>95th	1.48	0.86, 2.61		
HDL (mmol/L)	0.72	0.32, 1.52	2.64	0.78, 14.6
HDL Percentile				
<10th	—	—		
>10th	0.72	0.43, 1.18		
TG (mmol/L)	1.75*	1.02, 3.09	2.35*	1.16, 5.03
TG Percentile				
<95th	—	—		
>95th	1.43	0.73, 2.90		
FBS (mmol/L)	1.19	0.76, 1.87	1.16	0.72, 1.88

## Discussion

The study revealed that more than half of the children and adolescents who were overweight or obese had elevated levels of uric acid. Moreover, individuals with a greater BMI were found to have a higher likelihood of developing hyperuricemia. These results align with the findings of a previous study conducted in Spain [18]. In contrast, the prevalence of hyperuricemia among children with overweight and obesity in our study was significantly higher compared to findings from other studies. For instance, in a Chinese study, the reported prevalence rates were 9.9% for overweight children and 18.9% for obese children [19]. This difference may be attributed to genetic or dietary factors. 

Existing literature consistently demonstrates a greater prevalence of hyperuricemia among individuals who are obese compared to the general population. Moreover, numerous studies have established a clear link between increased body mass index (BMI) and elevated levels of uric acid. For instance, in Japan, research revealed that 42% of children and adolescents diagnosed with hyperuricemia also had metabolic syndrome, further emphasizing the association between these conditions [20]. Similarly, a study conducted on 2335 Brazilian schoolchildren revealed that hyperuricemia was more prevalent among those who had obesity and low levels of cardiorespiratory fitness [21]. In a similar vein, an Italian study focusing on children and adolescents found that individuals in the highest quartile of uric acid levels exhibited higher body weight and demonstrated poorer lipid and insulin metabolism profiles [22]. 

The relationship between uric acid levels and obesity is complex and multifaceted, with several theories proposed to explain this association [23]. One theory suggests that increased uric acid production can result from factors such as overnutrition and metabolic stress, where a higher intake of purines found in foods like red meat, organ meats, and certain seafood leads to elevated levels. Additionally, obesity is often linked to insulin resistance, which may further boost uric acid production. There may also be an upregulation of dihydrofolate reductase activity, an enzyme involved in purine synthesis, in individuals with obesity.

Another aspect involves decreased uric acid excretion. Obesity can lead to renal dysfunction, impairing the kidneys' ability to excrete uric acid effectively. Furthermore, abnormalities in uric acid transporters, such as URAT1 and ABCG2, may arise with obesity, hindering proper excretion. Increased reabsorption of uric acid in the distal nephron is also a concern in obese individuals.

Adipose tissue itself can be a source of uric acid, as adipocytes are capable of producing it, which contributes to elevated serum levels. The chronic inflammation and oxidative stress associated with obesity may further stimulate uric acid production in adipose tissue.

Finally, shared risk factors can play a significant role in this relationship. Certain genetic predispositions may make individuals more susceptible to both obesity and hyperuricemia. Lifestyle factors, including sedentary behavior, poor dietary choices, and excessive alcohol consumption, can also contribute to both conditions, reinforcing the connection between elevated uric acid levels and obesity.

Furthermore, elevated levels of uric acid play a significant role in the development of metabolic syndrome, and this influence can manifest as early as adolescence. This suggests that uric acid levels may serve as a useful tool in identifying and treating individuals at high risk for metabolic syndrome at an earlier stage [22].

Numerous studies have identified a significant association between elevated lipid levels and hyperuricemia [24-26]. While the exact mechanisms underlying this relationship are still being investigated, it is clear that these two conditions share underlying pathophysiological mechanisms. The underlying mechanisms include insulin resistance, which is characterized by the body's inability to effectively utilize insulin, leading to increased uric acid production and decreased excretion, as well as abnormal lipid metabolism. Chronic inflammation is another factor, as both hyperuricemia and dyslipidemia are associated with inflammatory processes. Uric acid can have pro-inflammatory effects, and elevated lipid levels can further promote inflammation, exacerbating both conditions. Additionally, renal dysfunction can occur in both hyperuricemia and dyslipidemia, impairing the kidneys' ability to excrete uric acid and regulate lipid metabolism. Genetic factors may also contribute to the development of both conditions, potentially explaining the observed association.

Hyperuricemia can serve as an early indicator of cardiovascular dysfunction. In fact, uric acid is recognized as an independent risk factor for heart failure and is linked to elevated cardiovascular mortality rates among both adults and children [22]. Furthermore, elevated levels of uric acid have demonstrated independent predictive capability for higher blood pressure levels [27].

Given that obesity is a significant risk factor for hyperuricemia, it is crucial to implement lifestyle interventions that encompass dietary modifications, increased physical activity, and behavioral changes aimed at weight reduction. These interventions play a vital role in managing hyperuricemia effectively.

In a study conducted by Togashi et al., it was shown that uric acid levels exhibited a significant decrease in a group of 33 obese children who underwent a treatment regimen consisting of diet modifications and exercise for a duration of three months [28]. Moreover, a weight reduction program spanning one year, targeting a cohort of 10 to 17-year-olds, was found to result in decreased uric acid levels in 86% of the females and 67% of the males [29]. While the impact of lifestyle intervention programs on obesity shows promise, further investigation is needed to assess their effect on hyperuricemia, particularly over an extended observation period.

One of the limitations of this study is the absence of a comparison group consisting of children and adolescents with normal BMI. This study only included overweight and obese individuals, which restricts the ability to directly compare the findings with those of the general population. In addition, the study established an association, but establishing causation requires additional evidence. Other possible confounding factors, like genetic and dietary factors, were not controlled in this study. It is difficult to determine if children and adolescents may have pre-existing elevated uric acid levels that have gone undetected.

## Conclusions

In conclusion, the study revealed a significant prevalence of hyperuricemia among children and adolescents who were overweight or obese, with an increased risk associated with male gender and elevated levels of low-density lipoproteins and triglycerides. The study highlights the potential of serum uric acid as a future biomarker for metabolic disorders. Additionally, further research is required to encompass children and adolescents across different weight categories, not solely those who are overweight or obese.

Moreover, the study underscores the significance of implementing lifestyle interventions such as dietary changes, increased physical activity, and behavioral modifications focused on weight reduction to effectively manage hyperuricemia.
